# Supplementary material for: Genetic associations with parental investment from conception to wealth inheritance in six cohorts
Source: Nat Hum Behav. 2023 Jun 29;7(8):1388–401. doi: 10.1038/s41562-023-01618-5 (PMC10444618; doi:10.1038/s41562-023-01618-5)
Supplement: Supplementary file 1 — Supplementary Methods, Figs. 1 and 2, Tables 1–4 and References. [file 41562_2023_1618_MOESM1_ESM.pdf]

nature human behaviour

Article https://doi.org/10.1038/s41562-023-01618-5

Genetic associations with parental investment from conception to wealth inheritance in six cohorts

In the format provided by the authors and unedited

Online-only material

Wertz et al., Genetic associations with parental investment from conception to wealth inheritance in six cohorts

SUPPLEMENTARY METHODS **2**
1. Description of cohorts **2**
1.1. The Environmental Risk (E-Risk) Longitudinal Twin Study 2
1.2. The Dunedin Study 3
1.3. The Avon Longitudinal Study of Parents and Children (ALSPAC) Study 3
1.4. The UK Millennium Cohort Study (MCS) 4
1.5. The Health and Retirement Study (HRS) 5
1.6. The Wisconsin Longitudinal Study (WLS) 5
2. Genotyping **5**
2.1. The E-Risk cohort 5
2.2. The Dunedin cohort 6
2.3. The ALSPAC cohort 6
2.4. The MCS cohort 6
2.5. The HRS cohort 7
2.6. The WLS cohort 7
3. Polygenic scoring **8**
4. Description of measures **8**
4.1. Prenatal period 8
4.2. Infancy 9
4.3. Childhood 9
4.4. Adolescence 13
4.5. Adulthood 14
5. Statistical analyses **15**
SUPPLEMENTARY FIGURES **17**
Supplementary Figure 1. ..... **17**
Supplementary Figure 2. ..... **19**
SUPPLEMENTARY TABLES **20**
Supplementary Table 1 ..... **20**
Supplementary Table 2 ..... **22**
Supplementary Table 3 ..... **23**
Supplementary Table 4 ..... **24**
SUPPLEMENTARY REFERENCES **26**

SUPPLEMENTARY METHODS

1. Description of cohorts

1.1. The Environmental Risk (E-Risk) Longitudinal Twin Study

The E-Risk Study tracks the development of a birth cohort of 2,232 British participants. The sample was drawn from a larger birth register of twins born in England and Wales in 1994-1995 [1]. Full details about the sample are reported elsewhere.[2] Briefly, the E-Risk sample was constructed in 1999-2000, when 1,116 families (93% of those eligible) with same-sex 5-year-old twins participated in home-visit assessments. This sample comprised 56% monozygotic (MZ) and 44% dizygotic (DZ) twin pairs; sex was evenly distributed within zygosity (49% male). Families were recruited to represent the UK population of families with newborns in the 1990s, on the basis of residential location throughout England and Wales and mother's age. Teenaged mothers with twins were over-selected to replace high-risk families who were selectively lost to the register through non-response. Older mothers having twins via assisted reproduction were under-selected to avoid an excess of well-educated older mothers. The study sample represents the full range of socioeconomic conditions in the UK, as reflected in the families' distribution on a neighbourhood-level socioeconomic index (ACORN [A Classification of Residential Neighbourhoods], developed by CACI Inc. for commercial use);[3] 25.6% of E-Risk families lived in "wealthy achiever" neighbourhoods compared to 25.3% nationwide; 5.3% vs. 11.6% lived in "urban prosperity" neighbourhoods; 29.6% vs. 26.9% lived in "comfortably off" neighbourhoods; 13.4% vs. 13.9% lived in "moderate means" neighbourhoods, and 26.1% vs. 20.7% lived in "hard-pressed" neighbourhoods. E-Risk underrepresents "urban prosperity" neighbourhoods because such households are likely to be childless.

Home-visits assessments took place when participants were aged 5, 7, 10, 12 and, most recently, 18 years, when 93% of the participants took part. At ages 5, 7, 10, and 12 years, assessments were carried out with participants as well as their mothers (or primary caretakers); the home visit at age 18 included interviews only with participants. Each twin was assessed by a different interviewer. These data are supplemented by searches of official records and by questionnaires that are mailed, as developmentally appropriate, to teachers, and co-informants nominated by participants themselves. The Joint South London and Maudsley and the Institute of Psychiatry Research Ethics Committee approved each phase of the study. Parents gave informed consent and twins gave assent between 5-12 years and then informed consent at age 18.

For our analyses the sample was restricted to participants of European-descent who had polygenic score data (n=880 mothers). This sample size reduces for individual analyses due to missing data; the n for each analysis is reported in the measures description below and in the respective Tables/Figures. Similarly, not all genotyped mothers also had genotyped children; thus, for our analyses including children's polygenic score, the sample size reduces to n= 860 genotyped mother-child dyads.

1.2. The Dunedin Study

Dunedin participants were members of the Dunedin Multidisciplinary Health and Development Study, a longitudinal investigation of health and behaviour in a birth cohort. Dunedin participants (N 1,037; 91% of eligible births; 52% male) were all individuals born between April 1972 and March 1973 in Dunedin, New Zealand, who were eligible on the basis of residence in the province and who participated in the first assessment at age 3. Full details about the sample are reported elsewhere.[4] The cohort represented the full range of socioeconomic status (SES) in the general population of New Zealand's South Island. On adult health, the cohort matches the New Zealand National Health and Nutrition Surveys on key health indicators (e.g., body mass index, smoking, visits to the doctor). Assessments with Dunedin participants were carried out at birth and ages 3, 5, 7, 9, 11, 13, 15, 18, 21, 26, 32, 38, and, most recently, 45 years. All but one of the assessments have enjoyed participation rates well above 90%.[4] The study was approved by the New Zealand Southern Health and Disability Ethics Committee and the Duke Campus Institutional Review Board. Written informed consent was obtained from all participants.

In 1994, when Dunedin participants were between 21 and 22 years old, a study of their parenting behaviour was initiated (the Parenting Study).[5] By 2017, when Dunedin participants were 44 – 45 years old, N 702 had participated in the parenting study, of N 738 cohort members eligible for participation based on their having a 3-year-old child (participation rate: 95%). For the majority of participants, the child they participated in the study with was their first-born (91%) biological child (97%). Dunedin study participant-parents and their children were visited in their home by an interviewer who conducted systematic observations of the home environment and who videotaped the parent interacting with his or her child. Children were observed when they were on average 3.3 years old, with 59% seen within 2 months of their third birthday (SD 0.5 years; range 2.1– 6.8 years). On average, parents were 33 years old at the time of the assessment (SD 5.7 years; range 21.5– 44.7 years). All dyad pairs (i.e., mother/son, mother/daughter, father/son, father/daughter) were equally represented.

For our analyses the sample was restricted to participants of European-descent who had polygenic score data (n=654; n=338 mothers and n=316 fathers). This sample size reduces for individual analyses due to missing data; the n for each analysis is reported in the measures' description below and in the respective Tables/Figures.

1.3. The Avon Longitudinal Study of Parents and Children (ALSPAC) Study

The Avon Longitudinal Study of Parents and Children (ALSPAC) is an ongoing longitudinal birth cohort study which started in the early 1990s. Recruitment and retention over time is described in detail elsewhere.[6,7] Briefly, all pregnant women living in Bristol, UK, and surrounding areas, with an expected delivery date between April 1, 1991 and Dec 31, 1992 were

eligible for inclusion. Of 14 541 pregnancies, 13 988 children were alive at 12 months. About 85% of eligible expectant mothers participated. When the oldest children were approximately 7 years of age, an attempt was made to bolster the initial sample with eligible cases who had failed to join the study originally, resulting in an additional 913 children being enrolled. Data collection was by postal questionnaires and regular 'focus' clinics, as previously described.[6,7] This study uses data collected up to age 16. Detailed information about ALSPAC is available online www.bris.ac.uk/alspac. The study website contains details of all the data that are available through a fully searchable data dictionary and variable search tool (http://www.bristol.ac.uk/alspac/researchers/our-data/). Ethical approval for the study was obtained from the ALSPAC Law and Ethics Committee and the Local Research Ethics Committees. Informed consent for the use of data collected via questionnaires and clinics was obtained from participants following the recommendations of the ALSPAC Ethics and Law Committee at the time. Consent for biological samples has been collected in accordance with the Human Tissue Act (2004). Each study was required to submit a research proposal to be approved by the executive committee before gaining access to the ALSPAC data. The overall aims of the study were included in this proposal.

For our analyses the sample was restricted to participants of European-descent who had polygenic score data (n=7,588 mothers). This sample size reduces for individual analyses due to missing data; the n for each analysis is reported in the measures description below and in the respective Tables/Figures. Similarly, not all genotyped mothers also had genotyped children; thus, for our analyses including children's polygenic score, the sample size reduces to n= 4,996 genotyped mother-child dyads.

1.4. The UK Millennium Cohort Study (MCS)

The MCS is an ongoing UK longitudinal birth cohort study that was set up to follow the lives of children born at the turn of the new century [8]. Recruitment and retention over time is described in detail elsewhere.[9] Briefly, children born between September 2000 and January 2002 across England, Scotland, Wales and Northern Ireland, alive and living in the UK at age 9 months were eligible for inclusion. Eligible children were identified using government child benefit records, a benefit with almost universal coverage. The sample contained 18 552 families (18 827 children) at baseline. The first sweep of data was collected when cohort members were around 9 months old and subsequent sweeps of data were collected at ages 3, 5, 7, 11, 14 and 17 years. At the age-14 assessment, saliva samples were collected for genotyping, from cohort members along with their biological mother and father if resident in the household and available. Parents provided written informed consent for all components of MCS. At the age 14 and 17 follow-ups, children also provided informed consent. The study website contains details of all the data that are available (https://cls.ucl.ac.uk/cls-studies/millennium-cohort-study/).

For our analyses the sample was restricted to participants of European-descent who had polygenic score data (n=6,700 mothers and n=3,613 fathers). This sample size reduces for individual analyses due to missing data; the n for each analysis is reported in the measures'

description below and in the respective Tables/Figures. Similarly, not all genotyped mothers/fathers also had genotyped children; thus, for our analyses including children's polygenic score, the sample size reduces to n= 5,421 genotyped mother-child dyads, n=2,903 genotyped father-child dyads and n=2,503 genotyped trios.

1.5. The Health and Retirement Study (HRS)

The HRS is a longitudinal survey of a representative sample of Americans aged >50 and their spouses, initiated in 1992 to study health and retirement among older people in the US.[10] HRS is administered biennially and includes over 26,000 persons in 17,000 households. During each wave of the survey, participants are asked about their economic well-being, health, social lives, and other factors relevant to aging and retirement (http://hrsonline.isr.umich.edu/index.php). The present study used data up to and including the 2016 survey. HRS was approved by the University of Michigan institutional review board and informed consent was obtained from each respondent.

For our analyses the sample was restricted to participants of European-descent who had polygenic score data (n= 8,652; n=5,052 female, n=3,600 male). This sample size reduces for individual analyses due to missing data; the n for each analysis is reported in the measures' description below and in the respective Tables/Figures.

1.6. The Wisconsin Longitudinal Study (WLS)

The Wisconsin Longitudinal Study (WLS) is based on a 1/3 random sample of all Wisconsin high school graduates in 1957 (N = 10,317) born between 1938 and 1940[11] and one of their randomly selected siblings (N=8,734). Waves of data collection from graduates (i.e., the primary respondents) or parents of graduates were conducted in 1957, 1964, 1975, 1992, 2003, and 2011 and from a sibling in 1977, 1993, 2004, and 2011. Ethical approval for analysis of the WLS genetic data was provided by the University of Cincinnati's Institutional Review Board.

For our analyses the sample was restricted to participants of European-descent who had polygenic score data (n=8,479; n=4403 female; n=4076 male). This sample size reduces for individual analyses due to missing data; the n for each analysis is reported in the measures' description below and in the respective Tables/Figures.

2. Genotyping

2.1. The E-Risk cohort

Genotyping of E-Risk cohort members and their mothers was performed using Illumina HumanOmni Express 24 BeadChip arrays (Versions 1.1 and 1.2 respectively; Illumina,

Hayward, CA). We imputed additional SNPs using the IMPUTE2 software (Version 2.3.1, https://mathgen.stats.ox.ac.uk/impute/impute_v2.html; Howie, Donnelly, & Marchini, 2009) and the 1000 Genomes Phase 3 reference panel [13]. Imputation was conducted on SNPs appearing in dbSNP (Version 140; http://www.ncbi.nlm.nih.gov/SNP/; Sherry et al., 2001) that were called in more than 98% of the samples. Invariant SNPs were excluded. The E-Risk cohort contains monozygotic twins, who are genetically identical; we therefore empirically measured genotypes of one randomly-selected twin per pair and assigned these data to their monozygotic co-twin. Prephasing and imputation were conducted using a 50-million-base-pair sliding window. The resulting genotype databases included genotyped SNPs and SNPs imputed with 90% probability of a specific genotype among European-descent members of the E-Risk cohort. We analysed SNPs in Hardy- Weinberg equilibrium ($p > .01$).

2.2. The Dunedin cohort

Genotyping of Dunedin cohort participant-parents was performed using Illumina HumanOmni Express 12 BeadChip arrays (Version 1.1; Illumina, Hayward, CA). We imputed additional SNPs using the IMPUTE2 software (Version 2.3.1, Howie et al., 2009) and the 1000 Genomes Phase 3 reference panel [13]. Imputation was conducted on SNPs appearing in dbSNP (Version 140; http://www.ncbi.nlm.nih.gov/SNP/; Sherry et al., 2001) that were called in more than 98% of the samples. Invariant SNPs were excluded. Prephasing and imputation were conducted using a 50-million base-pair sliding window. The resulting genotype databases included genotyped SNPs and SNPs imputed with 90% probability of a specific genotype among European-descent members of the Dunedin cohort. We analysed SNPs in Hardy- Weinberg equilibrium ($p > .01$).

2.3. The ALSPAC cohort

Genotyping of ALSPAC cohort members was performed using the Illumina HumanHap550 quad chip genotyping platforms by 23andme subcontracting the Wellcome Trust Sanger Institute, Cambridge, UK and the Laboratory Corporation of America, Burlington, NC, US. ALSPAC mothers were genotyped using Illumina human660w quad array at the Centre National de Genotypage (CNG) and genotypes were called with Illumina GenomeStudio. Quality control filtering was done using the PLINK (v1.07) software. SNPs with a minor allele frequency of < 1%, call rate < 95% and Hardy-Weinberg equilibrium (HWE) $P < 5E\text{-}7$ were removed. Both offspring and maternal genotype data have been jointly imputed to the 1000 genomes reference panel (version 1, Phase 3, Dec 2013 Release). All individuals with non-European ancestry were removed.

2.4. The MCS cohort

Genotyping of MCS cohort members and their biological parents was performed using Illumina Infinium global screening arrays-24 v1.0 in the Illumina Array Facility, University of Bristol. Genotypes were called with Illumina Genome Studio v2.0.4. Quality control was done using QCtools_v2.0.1 (https://www.well.ox.ac.uk/~gav/qctool/) and PLINK, using standard procedures described in detail elsewhere [15]. SNPs with a minor allele frequency of < 1%, call rate < 95% and Hardy-Weinberg equilibrium P < 2.5E-8 were removed. Imputation was done using the Michigan Imputation Server (MIS; imputationserver.sph.umich.edu). Prepared data was submitted to the MIS, phased with Eagle.v2.4 (Loh et al., 2016) and imputed to Haplotype Reference Consortium release 1.1 (HRC r1.1; http://www.haplotypereference-consortium.org) (McCarthy et al., 2016).

2.5. The HRS cohort

Genotyping of HRS cohort members was performed using the llumina HumanOmni2.5 BeadChips (HumanOmni2.5-4v1, HumanOmni2.5-8v1), at the Center for Inherited Disease Research (CIDR) at Johns Hopkins University. Individuals with missing call rates >2%, SNPs with call rates < 0.0001, chromosomal anomalies, and first degree relatives in the HRS were removed. Imputation was performed by the University of Washington Genetics Analysis Center (GAC) to the 1000 Genomes Project cosmopolitan reference panel phase 3 version 5 (initial release on May 2013, haplotypes released Oct 2014), using IMPUTE2.[12]

2.6. The WLS cohort

Genotyping of WLS cohort members was performed using the Illumina HumanOmniExpress array, at the Center for Inherited Disease Research (CIDR) at Johns Hopkins University and using the calling algorithm GenomeStudio version 2011.1, Genotyping Module version 1.9.4, GenTrain Version 1.0. Imputation was performed by the University of Washington Genetics Analysis Center (GAC), after quality control using the GAC's standardized QC procedures,[16] against the Haplotype Reference Consortium (HRC) v1.1 European reference panel[17] using IMPUTE2.[12]

3. Polygenic scoring

We computed polygenic scores based on the most recent Social Science Genetic Association Consortium (SSGAC) GWAS of educational attainment[18] (https://www.thessgac.org/data). In the E-Risk, Dunedin, ALSPAC and MCS cohorts, polygenic scores were calculated following the method described by Dudbridge[19] using the PRsice software [v1.22, http://prsice.info/; Euesden, Lewis, & O'Reilly, 2015)]. Briefly, SNPs reported in the GWAS[18] were matched with SNPs in each cohort, regardless of nominal significance for their association with educational attainment. We performed clumping by retaining the SNP with the smallest p value from each linkage disequilibrium block (excluding SNPs with r2>.1 in 500-kb windows), then weighted SNPs by effect estimate, and then summed weighted counts across all genotypes to calculate each participant's polygenic score.

In the HRS and WLS cohorts polygenic scores were computed by the SSGAC using the LD Pred software.[21] Because HRS and WLS data were included in the GWAS of educational attainment, polygenic scores for these datasets were constructed using summary statistics after the target dataset was excluded. In the current analysis, we rely on the polygenic score constructed using the multi-trait analysis of genome-wide summary statistics (MTAG; Turley et al., 2018) because it has been shown to improve the predictive power of polygenic scores.

Polygenic scoring in each cohort was restricted to individuals of European-ancestry. To account for potential population stratification, polygenic scores were residualised on the first ten principal components computed from the genome-wide data in each cohort.[23] Residualised polygenic scores were normally distributed and standardized to M=0, SD=1 in each cohort.

4. Description of measures

4.1. Prenatal period

Cigarette smoking. In E-Risk, mothers' smoking was assessed retrospectively, 1 year after birth, by asking whether mothers had smoked any cigarettes during the pregnancy (27.3%, total n=846). In ALSPAC, mothers' smoking was assessed prospectively, at 18 and 32 weeks of gestation, by asking whether mothers had smoked in the last two weeks (at 18 weeks gestation) or were currently smoking (at 32 weeks gestation). We combined data from both time points, replacing missing data at one time point with valid data from the other if available, to construct a measure of whether mothers reported smoking during pregnancy (19.7%; total n=7,190). In MCS, mothers' smoking was assessed retrospectively, at 9 months, by asking mothers about their history of smoking and about changes in smoking during pregnancy. We combined these questions following previous publications[24] to create a measure of whether mothers had reported smoking during pregnancy (21.0%, total n=6,690).

Heavy alcohol drinking. In ALSPAC, mothers' heavy drinking was assessed prospectively, at 18 and 32 weeks of gestation, by asking mothers how many days in the past month they had been drinking the equivalent of 4 units of alcohol (e.g. 2 pints of beer, 4 glasses of wine or 4 pub measures of spirit). We combined the response options ranging from "everyday" to "1-2 days" versus the response option "None". We combined data from both time points, replacing missing data at one time point with valid data from the other if available, to classify women as heavy drinkers (21.9%, total n=7,144). In MCS, mothers' heavy drinking was assessed retrospectively, at 9 months, by asking mothers whether they drank alcohol during pregnancy. Mothers who reported drinking regularly (between daily and once or twice a week when pregnant) were asked how many units of alcohol they drank per week on average. We divided the reported units of drinking by the reported weekly frequency of drinking to obtain an approximate measure of units consumed on days they drank (e.g., if a mother reported drinking every day, and reported drinking 20 units per week on average, it would be 20/7=2.86 units per day). Mothers who reported drinking rarely (1-2 times per month to less than once per month) were asked how many units of alcohol they drank on days they drank. We combined these two measures and categorized everyone who reported drinking 4 or more units on days they drank as heavy drinkers (2.7%, total n= 6,695).

4.2. Infancy

Breastfeeding. In E-Risk, breastfeeding was assessed when the children were 2 years old by asking whether mothers had ever breastfed (48.1%; total n=855; note that E-Risk is a twin sample, so breastfeeding rates would be expected to be lower than in singletons). In ALSPAC, breastfeeding was assessed when children were 4 weeks, 6 months and 15 months old, by asking mothers about their breastfeeding. We constructed a summary measure, replacing missing data at one time point with valid data from the other if available, indicating whether mothers had reported ever breastfeeding (75.6%; n=total n=7,025). In MCS, breastfeeding was assessed when the children were 9 months old by asking whether mothers had ever breastfed (73%, total n= 6,222).

4.3. Childhood

Cognitive stimulation. In E-Risk, cognitive stimulation was measured when children were aged 5, 7, 10, and 12 years, as previously described.[25] Briefly, at age 5, mothers responded to 12 items asking about activities with their twins (example items: "Have you and the twins visited a museum?"). The internal consistency reliability was α = .59. At ages 7, 10 and 12, study interviewers provided observations of each family's home using six items adapted from the Home Observation for Measurement of the Environment (HOME; Bradley, Caldwell, Rock, Hamrick, & Harris, 1988; Caldwell & Bradley, 1984) (example item: "Do the children have books?"). Internal consistency reliabilities ranged from α = .70 to α = .81 across ages (mean α =

.75) Measures were standardized within age, then averaged across age (total n=879). In Dunedin, cognitive stimulation was assessed using video-observations and home-observations as previously described [5,28]. Briefly, during the home visit, each parent– child dyad was videotaped in three, increasingly demanding, semi-structured situations, each lasting 10 min. Each situation was rated by trained coders using a set of 7-point scales developed for the NICHD Study of Early Child Care (NICHD Early Child Care Research Network, 1999). Interrater agreement ranged from .77 to .96. Following the home visit, interviewers rated each family on the HOME[27] including on items capturing the availability of learning materials and attempts by parents to teach skills (example item: “Child has three or more books of his or her own”). To construct a summary variable of cognitive stimulation that combines video-and home observations we standardized each measure and averaged them (total n=643). In ALSPAC, cognitive stimulation was assessed using three items that mothers were asked repeatedly (seven times each) between child age 6 months and 7 years: how often the child was taken out to the library, how often the mother read to the child, and the number of books the children owned. We constructed cross-age measures for each question, by standardizing within age and averaging across age (i.e. average visits to library across ages; average reading to the child across ages; average number of books owned by child across ages). We then standardized each cross-age measure and averaged them to create an overall summary variable of cognitive stimulation (total n=6,180). In MCS, cognitive stimulation was assessed at ages 5 and 7. At both ages, parents were asked about their reading with the child (“How often do you read to <child>”) and visits to the library (“Over the past 12 months, how often has <child> been to the library?”). We constructed cross-age measures for each question by averaging across age. We standardized each cross-age measure and averaged them to create an overall summary variable of cognitive stimulation (total n=6,077).

Warm, sensitive parenting. In E-Risk, warm, sensitive parenting was assessed when children were aged 5, 7, and 10 years as previously described.[25] Briefly, at ages 5 and 10, maternal warmth and dissatisfaction were each assessed using a 5 min speech sample from mothers.[30,31] Interrater agreement was r=.90 for maternal warmth and r=.84 for dissatisfaction. At ages 7 and 10, positive and negative parenting was assessed through study interviewer observations of parent–child interactions during the study visit using items adapted from the HOME[26,27] and the Dyadic Parent–Child Interactive Coding System–Revised[32,33] (example items: “Is the parent affectionate to the child?”; “Is the parenting of the child overly strict?”. Internal consistency reliabilities ranged from α = .72 to α = .82 (mean α = .82 for positive parenting, and α = .75 for negative parenting). Measures were standardized and averaged within age, then re-standardized and averaged across age (n=880). In Dunedin, warm, sensitive parenting was assessed as previously described,[5,28] using video-observations and home-observations as described for the measure of cognitive stimulation. Warm, sensitive parenting reflects parental expressions of warmth, affection and sensitivity toward their child (example items: “Parent’s voice conveys positive feelings towards child”; “Parent does not express overt annoyance with or hostility to child”). We standardized each measure and averaged them to construct a summary variable of

cognitive stimulation that combines video-and home observations (n=640). In ALSPAC, warm, sensitive parenting was assessed when children were 4, 7 and 10 years old, as previously described [34]. Briefly, at ages 4 and 7 mothers responded to 8 statements capturing positivity about the child (example item: “I really love this child”) and negativity (“I often get very irritated with this child”). Because the scores for positivity are heavily skewed, previous publications have used only the negativity scales;[34] we followed this same approach. Internal consistency reliabilities were α=.63 at age 4 and α=.71 at age 7. At age 10, children responded to 8 questions about their relationship with their parents (example item: “My parents like me”). Because of the highly skewed distributions of these items, we recoded them into binary items, combining response options 1 (not true); 2 (mostly untrue) and 3 (partly true) versus 4 (mostly true) and 5 (true). We then averaged across items to create a summary score. Internal-consistency reliability was α=.71. We standardized each of these measures within age and then averaged across ages to construct a cross-age summary measure of warmth and sensitivity (n=6,324). In MCS, warm, sensitive parenting was assessed at ages 3, 7 and 11. At age 3, study interviewers rated parent–child interactions during the study visit using binary items adapted from the HOME[26,27] (example items: “Mother's voice is positive when speaking to <child>”). We averaged across items. Because the resulting measure was so skewed in this sample, we recoded it to be binary, so that participants who had scored positively in each item received a 1, and participants who had scored anything less received a 0. At age 3, parents also responded to 15 items from the Child-Parent Relationship Scale (CPRS; Driscoll & Pianta, 2011; example items: “I share an affectionate, warm relationship with <child>”; “<Child> and I always seem to be struggling with each other”). Previous analyses by the MCS team [35] report internal consistency reliabilities of α=.83 for the conflict subscale and α=.72 for the closeness subscale. We used a previously-constructed measure, supplied by MCS, for our analyses. We constructed an age-3 summary measure by standardizing the HOME and CPRS measures and averaging across measures. At age 7 and 11, parents responded to 5 items (at age 7) and 2 items (age 11) asking about the relationship with their child (example item: “I have frequent battles of will with <child>”). At each age, we averaged across items to create summary measures. We then standardized each measure within age and averaged across ages to construct an overall summary measure (total n= 6,553).

Household chaos. In E-Risk, household chaos was assessed when children were aged 7, 10, and 12 years as previously described [25]. Briefly, at ages 7, 10, and 12, household chaos was assessed through study interviewers’ observations of family’s homes using three items adapted from the HOME (Bradley et al., 1988; Caldwell & Bradley, 1984; example item: “Is the house chaotic or overly noisy?”). Internal consistency reliabilities ranged from α = .53 to α = .58 across ages (mean α = .56). At age 12, household chaos was assessed through reports from mothers and children using 12 items adapted from the Confusion, Hubbub, and Order Scale (CHAOS; Matheny, Wachs, Ludwig, & Phillips, 1995), the Family Routines Inventory[37] and the Family Ritual Questionnaire [38] following previous research (Evans, Gonnella, Marcynyszyn, Gentile, & Salpekar, 2005; example items: “You can hardly hear yourself think in our home”; “We are

always losing things at home”). Internal consistency reliabilities were α = .76 for mother’s report and α = .78 for children’s report. Measures were standardized and averaged within age, then re-standardized and averaged across age (n=878). In ALSPAC, household chaos was assessed between 18 months and 10 years using items that asked about routines, noise and crowding in the home. At 18 months, 2.5 years, 5 years, 6 years, 7 and 9 years mothers responded to 1 item about whether their child had a regular sleep routine. We constructed a summary measure by averaging across ages. At 10 years, mothers responded to three questions asking about the noise level in their home (example item: “It is often so noisy at home it is difficult to hold a conversation”). At age 2 and 2.5 years, we used a previously-constructed household crowding that was based on maternal reports of the number of persons living in the home [40]. To construct a summary measure, we standardized each individual measure, then averaged across measures (n=6,210). In MCS, household chaos was assessed when children were 3, 5, 7 and 11 years old. At each age, mothers responded to 1-2 items about whether children had set routines (example item: ”Child has regular bedtimes”). We constructed a summary measure by averaging across ages. At age 3 and 11, mothers additionally responded to items about the atmosphere at home (example item: “You can hardly hear yourself think in our home”). We constructed a summary measure by averaging across ages. We then standardized these individual measure and averaged across measures to construct an overall summary measure (total n=6,615).

Health-parenting (parents’ promotion of a healthy lifestyle for their children). In E-Risk, health-parenting was assessed at the age-10 home visit as previously described,[41] using mother’s responses to items about children’s health-related behaviours, including questions about how much time children spent watching TV; their diet; and their tooth-brushing (example items: “How many hours of television do the twins watch on an average day?”). We averaged across items to create a summary measure (n=877). In ALSPAC, health-parenting was assessed between 15 months and 13 years, using three sets of items that were repeatedly asked across ages (six times each), asking about how much time children spent watching TV; their diet; and their tooth-brushing. For TV watching, mothers responded to a question about how many hours their child spent watching TV on weekdays and weekends; we combined answers to both questions. For tooth brushing, mothers responded to a question about the frequency that their child brushed teeth; we categorized responses into “once or less than once per day” and “more than once per day”. For diet, mothers responded to questions about how often their child ate a range of foods. These variables have previously been factor-analysed to derive dietary patterns, including ‘processed’ and ‘health conscious’ diets.[42] To construct a summary measure of health-parenting, we averaged each set of items (i.e. TV watching; tooth brushing; processed diet; health-conscious diet) across ages, standardized the cross-age measures, and then averaged across these measures (n=5,649). In MCS, health-parenting was assessed at ages 3, 5, 7 and 11. At each age, mothers responded to an item asking how many hours children spent watching TV per day. We averaged these items across ages. At ages 5, 7 and 11, mothers additionally responded to items asking about children’s diet, such as how many portions of fruit the child ate per day. We

averaged these items across ages. We then standardized these individual measures and averaged across measures to construct an overall summary measure (n=6,437).

School support. In ALSPAC, parents' school support (engagement with schooling and ambitions for children's education) was assessed between child ages 7 and 12 years, using mother and teacher reports. Mothers were asked at three ages (7, 10 and 11 years) about their help with schoolwork (variations of the item "Mum helps child prepare for school"). Mothers were also asked (at 10, 11, and 12 years) about their hopes for their child's schooling (variations of the item "What sort of education do you hope your child will have?" with response options ranging from "the minimum – and leave school as soon as possible" to "to go to University"). Teachers were asked at two ages (7-8 years and 10-11 years) how supportive the teacher thought the parents are towards the child's learning and how involved the parents were in the child's schooling (example items: "Child's parents have attended teacher parent session"; "Childs parents have been involved in other school activities"). We constructed cross-age measures by averaging across ages, then standardized each measure and averaging across measures to create an overall summary measure (n= 6,603). In MCS, parents' school support was assessed at ages 7 and 11, using mother, child and teacher reports. At both ages, mothers responded to questions about their hopes for their child's schooling (example item: "What would you like <child> to do at the age of 16?"). At both ages, mothers were also asked about their involvement with schooling (example item: "During this school year has anyone at home been to a parents' evening or similar event at school?"). At age 11, mothers were asked about their help with schoolwork (example item: "How often does anyone at home make sure [child's] homework is complete?"). At age 11, teachers and children were asked how interested parents were in children's education (example item: "How often do your parents take an interest in your school work?"). We constructed cross-age measures by averaging across ages, then standardized each measure and averaging across measures to create an overall summary measure (n= 6,587).

4.4. Adolescence

Parental monitoring (*rule-setting and knowledge of children's activities and whereabouts*). In E-Risk, parental monitoring was assessed at age 12 as previously described.[43] Briefly, at age 12 mothers and children each responded to ten items adapted from the Monitoring and Supervision Questionnaire[44] (example items "Do you know where <name> goes during his/her free time?"; "Does <name> need to have your permission to leave home (or go somewhere with friends)?". Internal consistency reliabilities were α =.66 for mothers' reports and α =.71 for children's reports. We constructed a summary measure by standardizing within informant and averaging across informants (n=866). In ALSPAC, parental monitoring was measured at child ages 13 and 14 (children's reports) and at child age 17 (mothers' reports). At child ages 13 and 14 parental monitoring was assessed using a computer-assisted survey completed by the young person when they attended a research clinic. Young people responded to

24 items (at age 13) and 10 items (at age 14) asking about parents' knowledge and monitoring[44] (example item: "How often do your carers / parents know what you do during your free time?"; "How often do you have to have your carers / parents' permission before you go out on weeknights?"). Internal-consistency reliability was α = .89 at age 13 and α = .84 at age 14. We averaged across items at each age. At age 17, mothers responded to 10 items asking about parental knowledge and monitoring[44] (example item: "When <child> went out during the last year, how often did you know what child was doing in their spare time?"; "During the past year, how often have you started a conversation with <child> about what they were doing in their spare time?". Internal-consistency reliability was α=.80. We averaged across items. To construct a summary measure of parental monitoring across ages, we standardized each measure within age and then averaged across ages (n=4,092). In MCS, parental monitoring was assessed at 14. At age 14, adolescents and mothers each responded to 3 questions about parental knowledge of adolescents' activities and whereabouts (example item: "When child goes out how often do you know where they are going?"). Internal consistency reliabilities were α=.76 for mothers' reports, and α=.81 for children's reports. To construct a summary measure of parental monitoring, we standardized each measure within informant and averaged across informants (total n=6,625).

4.5. Adulthood

Financial help provided to offspring. In HRS, financial help provided to offspring was assessed by asking participants if they had given financial help totaling $500 or more to any of their children or grandchildren since last interview (1: Yes; 0: No) (n=8,403). In WLS, financial help was assessed by asking participants if they or their spouse had given anyone a total of $1,000 or more in money, property or other assets (including money for a down payment on a home, living expenses, to pay for education, medical care, or for other needs) since the last interview (1: Yes; 0: No). Anyone who responded yes was asked a follow-up question, about whether these gifts were given to respondents' children. All respondents who responded yes to both questions received a 1 and 0 otherwise (n=8,082).

Support with childcare. In HRS, support with childcare was assessed by asking participants whether they had spent 100 hours or more taking care of grandchildren or great grandchildren since the last interview (n=7,451). In WLS, support with childcare was assessed by asking participants a series of four questions that began, "During the past month, did you help your sons or daughters who are 19 or older with…". These questions concluded with different types of support respondents might provide for their children, including "babysitting or childcare". We coded everyone who responded 'yes' to babysitting or childcare as 1 and 0 otherwise (n=5,624).

Leaving an inheritance. In HRS, the probability of leaving an inheritance was assessed by asking HRS participants about the chance that they would leave an inheritance totaling

$10,000 or more, with response options ranging from 0 (Absolutely no chance) to 100 (Absolutely certain) (n=8,626). In WLS, the intention of leaving an inheritance was assessed by asking participants about who would get their assets, including home, savings, life insurance and the like, if they were to die tomorrow, and then (if respondents reported having a spouse), who would get their assets if they outlived their spouse. Responses were open-ended, thus allowing respondents to designate anyone of any relation. We coded responses that included biological sons or daughters as 1 and 0 otherwise. Many respondents (i.e., 41% in 2003-07 and 59% in 2011) replied with "spouse" to the first question, thus we relied on responses from the second question. For respondents who reported not currently having a spouse, responses from the first question were used. Thus, the interpretation of this variable is that it indicates the intention of leaving an inheritance to *only* one's children (i.e., as opposed to every other reported combination of relations). Responses from both groups were combined, producing a single dichotomous item (n=7,217).

5. Statistical analyses

To analyse binary outcomes, we used Poisson regressions and report relative risks. To present these analyses visually, we used marginsplots as implemented in Stata. Each margins plot reports the predicted probabilities of the outcome at each level of the polygenic score. To analyse continuous outcomes, we used linear regressions and report standardized regression coefficients. To present these analyses visually, we used forest plots. Each forest plot reports a meta-analysed estimate across cohorts, as obtained using a random-effects model. All significance tests were two-tailed. Analyses of the ALSPAC, E-Risk, Dunedin, and MCS cohorts were conducted using Stata version 17.0,[45] as well as Mplus version 8.2 for E-Risk;[46] analyses of WLS and HRS were conducted using R. Because E-Risk is a twin sample, we used structural equation models for dyads with indistinguishable members to take into account the unique structure of the data.[47] Because the MCS cohort has a complex stratified and clustered design and non-random dropout over the years, we used sampling weights that correct for design and nonresponse, as well as adjustment for clustering, following instructions published by the MCS Research Team.[48] Following these instructions, when analysing longitudinal data, we used the weight for the last time point that was included in the construction of the outcome variable (e.g. if data up to age 14 was included, we used the weight provided for age 14).

In models predicting childhood and adolescent parenting, we adjusted for child sex. In models predicting parental investment to adult children, we also adjusted for parents' age, net worth (in WLS) or assets (in HRS), number of children, labour force status, and, for analyses predicting help with childcare, physical proximity to offspring.

We dealt with missing data in the construction of measures using a "60%" rule: participants needed to have valid data in at least 60% of time points across age, in order to be included in a measure. For example, for constructing a cross-age measure of "visits to the library" across 7 time points in ALSPAC, those with 3 or less missing data points were included. For aggregating these individual measures, all available data were used. For example, for

constructing an aggregate measure of cognitive stimulation in ALSPAC, made up of cross-age measures for visits to the library, reading with the child, and books the child owned, we included participants with valid data in at least one of these three cross-age measures (participants with missing data in all three measures were excluded).

We dealt with missing data in our analyses by including participants who had valid data on all measures (constructed as described above). In ALSPAC, E-Risk, Dunedin and MCS we conducted sensitivity analyses using Full Information Maximum Likelihood (FIML) estimation as implemented in Stata; this did not change the results. The exact n for each measure is reported in the measures' description.

The premise and analysis plan for this project were pre-registered at https://sites.duke.edu/moffittcaspiprojects/files/2021/07/Wertz_2019a.pdf. All analyses reported here were checked for reproducibility by an independent data-analyst, who recreated the code by working from the manuscript and applied it to a fresh dataset.

SUPPLEMENTARY FIGURES

Supplementary Figure 1. Associations between maternal and child polygenic scores and childhood parenting in the ALSPAC, E-Risk and MCS cohorts.

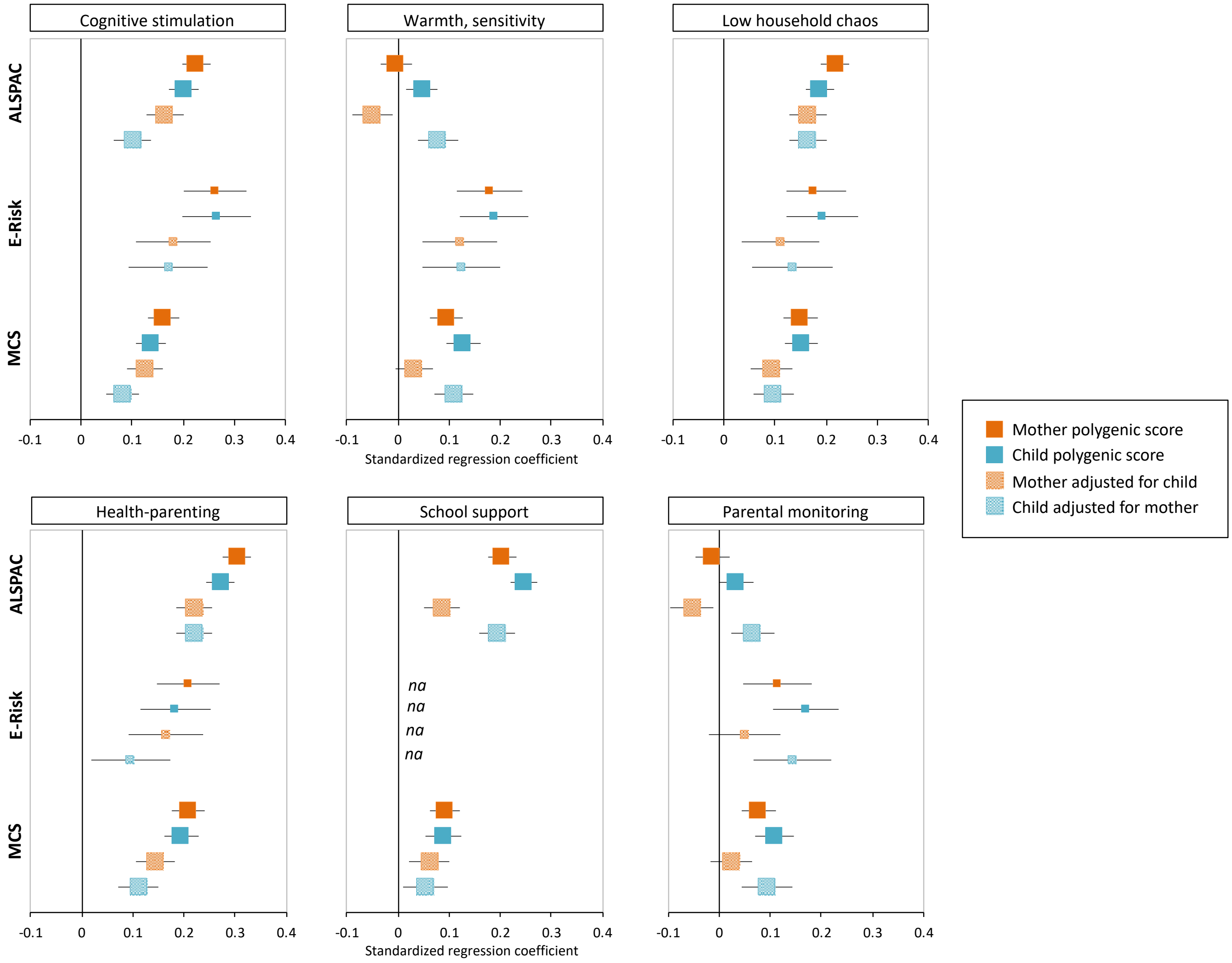

Note: The Figure shows associations (expressed as standardized regression coefficients) between maternal and child education polygenic scores and measures of parenting during childhood (cognitive stimulation; warmth, sensitivity; low household chaos; health-parenting; school support) and adolescence (parental monitoring) in the ALSPAC, E-Risk, and MCS cohorts (the Dunedin cohort is not included because it does not contain measures of child genetics). Orange boxes indicate mother polygenic scores, before (darker orange) and after (patterned orange) adjusting for child polygenic score. Blue boxes indicate child polygenic scores, before (darker blue) and after (patterned blue) adjusting for mother polygenic score. Not all measures were available in each cohort (e.g. measures of school support were only available in the ALSPAC

and MCS cohorts). The centre of the effect marker indicates the estimate of the association between polygenic score and parenting, expressed as a standardized regression coefficient. The error bars indicate 95% confidence intervals. The size of the effect size markers corresponds to the sample size, so that larger sample sizes have larger markers. The number of participants (mother-child dyads) included in the analysis were as follows: for cognitive simulation ALSPAC n=4,342; E-Risk n=859; MCS n=5,093; for warmth, sensitivity ALSPAC n=3,926; E-Risk n=860; MCS n=5,225; for low household chaos ALSPAC n=4,451; E-Risk n=858; MCS n=5,117; for health-parenting ALSPAC n=4,093; E-Risk n=858; MCS n=5,124; for school support ALSPAC n=4,586; MCS n=5,228; for parental monitoring ALSPAC n=3,343; E-Risk n=847; MCS n=5,414. ALSPAC=Avon Longitudinal Study of Parents and Children; E-Risk=Environmental Risk Longitudinal Twin Study; MCS=Millennium Cohort Study.

Supplementary Figure 2. Associations between mothers', father's and child polygenic scores in the MCS cohort.

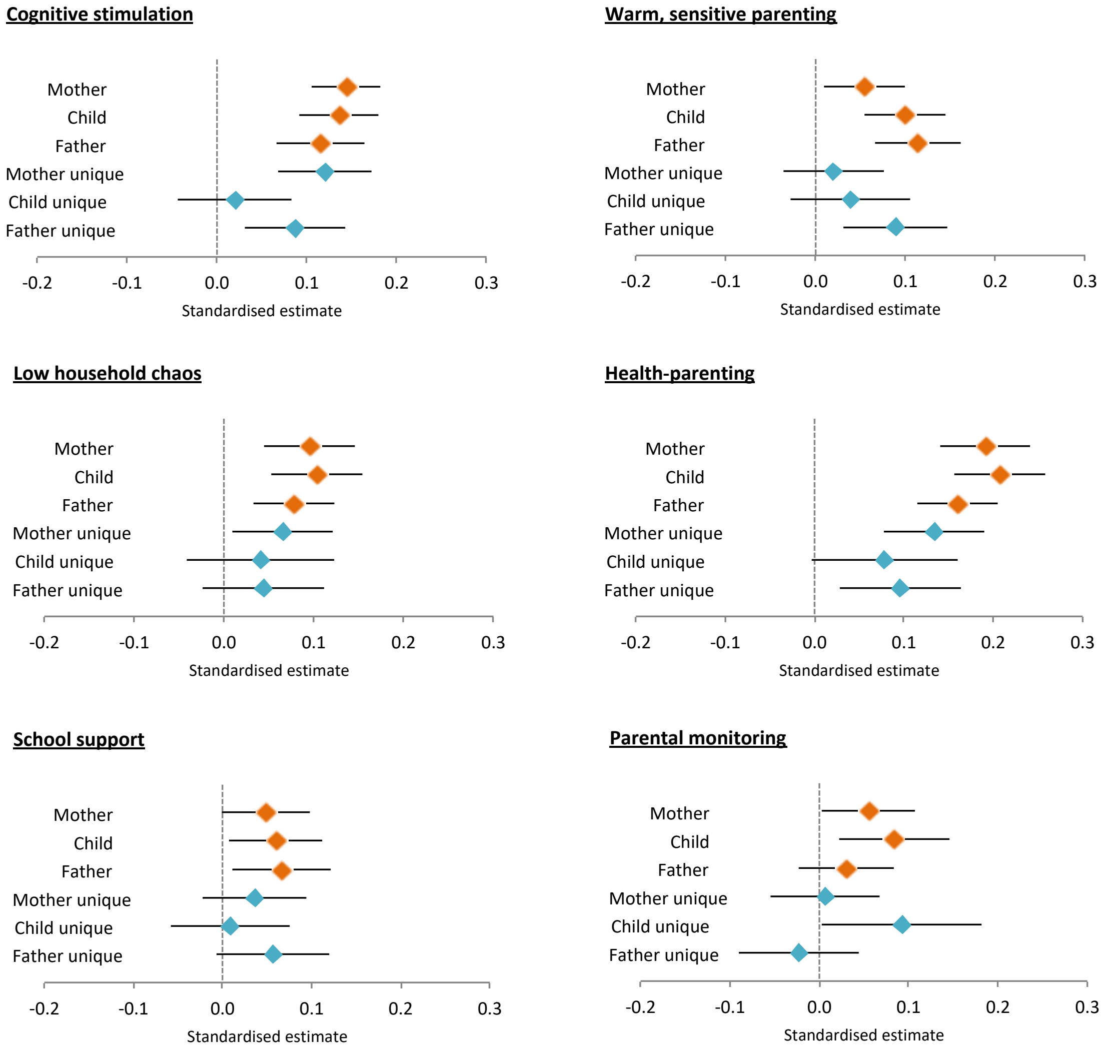

Note: The figure shows standardised estimates of associations between mother, father and child education polygenic scores and parenting, during childhood and adolescence, both for mother, father and child polygenic scores individually (in orange) as well as the unique association for each score when in a model containing adjusting for the others (in blue). The centre of the effect marker indicates the estimate of the association between polygenic score and parenting, expressed as a standardized regression coefficient. The error bars indicate 95% confidence intervals. All analyses were done in the subset of MCS participants who had genetic data and parenting data (n=2,503; with slightly lower n's across parenting measures).

SUPPLEMENTARY TABLES

Supplementary Table 1. Evidence from previous research for associations between parental investment and child outcomes and for associations between children's genes and child outcomes.

**Evidence for associations between parental investment and child outcomes**	
Developmental period	Description of evidence
Prenatal period	Many observational studies report associations between prenatal smoking and heavy drinking and various child outcomes, including physical health outcomes (such as birth weight, BMI, asthma),[49–51] behaviour[52] and cognition.[53] Although most of these studies control for confounders, they may still suffer from residual confounding, including from genetic influences. Evidence from RCTs or natural experiments, including genetically-sensitive designs, suggests effects of prenatal smoking predominantly on birth weight.[54–56] Likewise, most of the evidence for links between prenatal heavy drinking and many adverse child outcomes comes from observational studies;[57,58] evidence from quasi-experimental studies suggests a potential causal role of prenatal alcohol exposure on cognitive outcomes, and weaker evidence for a role in low birthweight.[59]
Infancy	As with prenatal smoking and heavy drinking, most of the evidence linking breastfeeding to child outcomes comes from observational studies. These studies show associations with many child outcomes, particularly childhood physical health outcomes such as obesity[60] and asthma,[61] and with child cognitive outcomes.[62] As with prenatal smoking and heavy drinking, a threat to the interpretation of these results is that observational studies may suffer from residual confounding. A review of evidence from different study designs, including experimental and quasi-experimental studies, suggests effects of breastfeeding on cognitive ability.[63]
Childhood	A wealth of observational evidence reports associations between various dimensions of parenting and child outcomes. We focused on dimensions of parenting that have been most commonly examined in these studies and that have been most consistently associated with a wide variety of outcomes; these parenting dimensions include cognitive stimulation,[64,65] warm-sensitive parenting,[66–68] household chaos,[69,70] health-parenting (i.e. parent efforts at instilling healthy habits in their children e.g. via limiting screen time or providing healthy foods),[71,72] and support with schooling.[73] These observational studies suffer from the same limitations as explained above, particularly the risk of residual confounding. However, there is some evidence from experimental and quasi-experimental designs to suggest a potential causal impact of these parenting dimensions for child outcomes, including evidence for effects of cognitive stimulation on child language outcomes,[74,75] warm-sensitive parenting on externalising

	problems,[76–78] household-chaos on externalising problems,[79] health-parenting on some child health outcomes,[80,81] and school support on academic achievement.[82,83]
Adolescence	One of the most well-researched aspects of parenting during adolescence is parental monitoring; numerous observational studies report associations between monitoring and offspring outcomes, particularly antisocial behaviour,[67] substance use and risky sexual behaviour,[84] and academic achievement.[68] Evidence from (quasi-)experimental research is more sparse, but suggests that parenting interventions during adolescence can reduce adolescents' risky substance-use and sexual behaviour.[85,86]
Offspring adulthood	We focus on three common sources of intergenerational supports from parents to adult offspring: financial support, wealth inheritance, and childcare support. Perhaps unsurprisingly, previous research suggests that financial support and wealth inheritance increase offspring wealth, at least in the short term.[87–89] For the provision of childcare support to the children of adult offspring, there is evidence from survey studies suggesting that it affects the labor market participation of mothers, as well as parents' fertility decisions.[90–92]
**Evidence for associations between children's genes and child outcomes**	
	Decades of evidence from twin and adoption studies show genetic influences on various offspring outcomes, including physical health, mental health, behavioural and educational outcomes.[93] More recent evidence for genetic influences comes from genome-wide association studies (GWAS) that have identified associations between measured genetic variation and various outcomes.[94] Findings from GWAS studies may suffer from several sources of confounding, such as indirect genetic effects, assortative mating or population stratification.[95] However, evidence from analyses of siblings (which control for potent sources of confounding) suggest that even among siblings born to the same biological parents, genetic differences continue to be associated with outcomes (although the magnitude of effects tends to reduce).[96]

Supplementary Table 2. Measurement of parental investment across cohorts.

	Child age at assessments	Informant	Format
Prenatal			
ALSPAC	18w, 32w	Mother	Questionnaire
E-Risk	2y	Mother	Questionnaire
MCS	9m	Mother	Questionnaire
Infancy			
ALSPAC	4wk, 6m, 1y	Mother	Questionnaire
E-Risk	2	Mother	Questionnaire
MCS	9m	Mother	Questionnaire
Childhood			
ALSPAC	1-12y	Mother	Questionnaire
E-Risk	5y,7y,10y,12y	Mother,<br>Interviewer	Questionnaires<br>HOME observations<br>Speech sample
MCS	3y,5y,7y,11y	Mother, Father,<br>Child, Teacher	Questionnaires
Dunedin	3y	Mother or Father,<br>Interviewer	Video observations<br>HOME observations
Adolescence			
ALSPAC	14y, 17y	Mother, Child	Questionnaire
E-Risk	12y	Mother, Child	Questionnaire
MCS	14y	Mother, Child	Questionnaire
Adulthood			
WLS cohort	na	Parent	Questionnaire
HRS cohort	na	Parent	Questionnaire

Note: ALSPAC = Avon Longitudinal Study of Parents and Children; E-Risk = Environmental Risk Longitudinal Twin Study; MCS = Millennium Cohort Study; HRS = Health and Retirement Study; WLS = Wisconsin Longitudinal Study

Supplementary Table 3. Associations between parental polygenic score and intergenerational supports to adult offspring across models with adjustment for different sets of variables.

	Health and Retirement Study (HRS)			
	Model 1	Model 2	Model 3	Model 4
	RR (95%CI)	RR (95%CI)	RR (95%CI)	RR (95%CI)
Financial support	1.12 [1.10; 1.14)	1.12 [1.10; 1.14]	1.11 [1.09; 1.13]	1.10 [1.08; 1.12]
Help with childcare	1.03 [1.01; 1.06]	1.04 [1.02; 1.07]	1.05 [1.02; 1.07]	1.04 [1.01; 1.06]
	β (95%CI)	β (95%CI)	β (95%CI)	β (95%CI)
Inheritance	0.12 [0.11; 0.14]	0.12 [0.11; 0.13]	0.12 [0.11; 0.13]	0.11 [0.10; 0.12]
	Wisconsin Longitudinal Study (WLS)			
	Model 1	Model 2	Model 3	Model 4
	RR (95%CI)	RR (95%CI)	RR (95%CI)	RR (95%CI)
Financial support	1.06 [1.04; 1.09]	1.07 [1.04; 1.09]	1.07 [1.04; 1.10]	1.07 [1.04; 1.09]
Help with childcare	1.10 [1.05; 1.14]	1.11 [1.07; 1.15]	1.11 [1.07; 1.16]	1.11 [1.07; 1.15]
Inheritance	1.00 [0.98; 1.02]	1.00 [0.98; 1.02]	1.00 [0.98; 1.02]	1.00 [0.98; 1.02]

Note: RR=Relative Risk; β=Standardized regression coefficient; CI=Confidence interval.
Model 1: Adjusted for wave/year, age, sex
Model 2: Adjusted for all the predictors as in Model 1, plus number of children (and, for childcare, proximity to children)
Model 3: Adjusted for all the predictors as in Model 2, plus labour force status
Model 4: Adjusted for all the predictors as in Model 2, plus assets/net worth

Supplementary Table 4. Associations between parental polygenic score and parenting, before (Model 1) and after (Model 2) adjusting for parental educational attainment.

	ALSPAC		E-Risk	
	Model 1*	Model 2^	Model 1	Model 2
Prenatal	RR (95%CI)	RR (95%CI)	RR (95%CI)	RR (95%CI)
Cigarette smoking	0.76 [0.72; 0.80]	0.91 [0.85; 0.97]	0.85 [0.75; 0.97]	0.96 [0.84; 1.11]
Heavy drinking	0.86 [0.82; 0.91]	0.93 [0.87; 0.98]	-	-
Infancy	RR (95%CI)	RR (95%CI)	RR (95%CI)	RR (95%CI)
Breastfeeding	1.11 [1.08; 1.14]	1.03 [1.00; 1.07]	1.24 [1.13; 1.37]	1.12 [1.01; 1.24]
Childhood	β (95%CI)	β (95%CI)	β (95%CI)	β (95%CI)
Cognitive stimulation	0.22 [0.20; 0.25]	0.08 [0.05; 0.10]	0.26 [0.20; 0.32]	0.09 [0.04; 0.15]
Warmth, sensitivity	-0.01 [-0.04; 0.02]	0.00 [-0.04; 0.03]	0.17 [0.11; 0.24]	0.07 [0.01; 0.14]
Household chaos	0.22 [0.19; 0.24]	0.09 [0.25; 0.30]	0.18 [0.11; 0.24]	0.04 [-0.02; 0.10]
Health-parenting	0.30 [0.27; 0.32]	0.14 [0.11; 0.17]	0.21 [0.14; 0.27]	0.07 [0.00; 0.13]
School support	0.20 [0.18; 0.23]	0.08 [0.05; 0.11]	-	-
Adolescence	β (95%CI)	β (95%CI)	β (95%CI)	β (95%CI)
Monitoring	-0.02 [-0.05; 0.02]	-0.05 [-0.08; -0.01]	0.11 [0.04; 0.17]	0.03 [-0.03; 0.10]
	HRS		WLS	
	Model 1	Model 2	Model 1	Model 2
Adulthood	RR (95%CI)	RR (95%CI)	RR (95%CI)	RR (95%CI)
Financial support	1.10 [1.08; 1.12]	1.04 [1.02; 1.06]	1.07 [1.04; 1.09]	1.01 [0.98; 1.04]
Childcare support	1.04 [1.01; 1.06]	1.01 [0.99; 1.04]	1.11 [1.07; 1.15]	1.08 [1.04; 1.12]
	β (95%CI)	β (95%CI)	RR (95%CI)	RR (95%CI)
Inheritance	0.11 [0.10; 0.12]	0.04 [0.03; 0.06]	1.00 [0.98; 1.02]	1.01 [0.99; 1.03]

Supplementary Table 4 continued

	Dunedin		MCS	
	Model 1	Model 2	Model 1	Model 2
Prenatal	RR (95%CI)	RR (95%CI)	RR (95%CI)	RR (95%CI)
Cigarette smoking	-	-	0.75 [0.71; 0.80]	0.93 [0.88; 0.99]
Alcohol drinking	-	-	0.97 [0.83; 1.12]	1.08 [0.91; 1.28]
Infancy	RR (95%CI)	RR (95%CI)	RR (95%CI)	RR (95%CI)
Breastfeeding	-	-	1.12 [1.10; 1.14]	1.05 [1.03; 1.07]
Childhood	β (95%CI)	β (95%CI)	β (95%CI)	β (95%CI)
Cognitive stimulation	0.11 [0.01; 0.22]	0.00 [-0.10; 0.10]	0.16 [0.13; 0.19]	0.07 [0.04; 0.11]
Warmth, sensitivity	0.15 [0.05; 0.26]	0.05 [-0.05; 0.15]	0.09 [0.05; 0.12]	0.03 [-0.01; 0.06]
Household chaos	-	-	0.14 [0.11; 0.17]	0.06 [0.02; 0.09]
Health-parenting	-	-	0.21 [0.18; 0.24]	0.08 [0.05; 0.11]
School support	-	-	0.09 [0.06; 0.12]	0.02 [-0.01; 0.06]
Adolescence	β (95%CI)	β (95%CI)	β (95%CI)	β (95%CI)
Monitoring	-	-	0.09 [0.05; 0.12]	0.04 [0.01; 0.08]

Note: ALSPAC=Avon Longitudinal Study of Parents and Children; E-Risk=Environmental Risk Longitudinal Twin Study; MCS=Millennium Cohort Study; HRS=Health and Retirement Study; WLS=Wisconsin Longitudinal Study. RR=Relative Risk; β=Standardized regression coefficient; CI=Confidence interval. Note, the N in these analyses was restricted to every parent who had valid data for polygenic score and parenting, as well as for educational attainment, so Ns (and estimates) may differ slightly from those in the main analyses.

* Model 1: Unadjusted for education (predictors are polygenic score, sex, and for WLS and HRS, age, wave/year, sex, number of children, physical proximity, labor force status and net worth/assets), these estimates might at times differ very slightly from those reported in the main manuscript, because some individuals included in the main analyses had available data for educational attainment.

^ Model 2: Adjusted for parental education (i.e. parental educational attainment is added to Model 1 as a predictor).

SUPPLEMENTARY REFERENCES

1. Trouton, A., Spinath, F. M. & Plomin, R. Twins early development study (TEDS): A multivariate, longitudinal genetic investigation of language, cognition and behavior problems in childhood. *Twin Res.* **5**, 444–448 (2002).
2. Moffitt, T. E. & E-Risk Study Team. Teen-aged mothers in contemporary Britain. *J. Child Psychol. Psychiatry* **43**, 727–42 (2002).
3. Odgers, C. L., Caspi, A., Bates, C. J., Sampson, R. J. & Moffitt, T. E. Systematic social observation of children's neighborhoods using Google Street View: A reliable and cost-effective method. *J. child Psychol. psychiatry* **53**, 1009–17 (2012).
4. Poulton, R., Moffitt, T. E. & Silva, P. A. The Dunedin Multidisciplinary Health and Development Study: Overview of the first 40 years, with an eye to the future. *Soc. Psychiatry Psychiatr. Epidemiol.* **50**, 679–93 (2015).
5. Belsky, J., Sligo, J., Jaffee, S. R., Woodward, L. & Silva, P. A. Intergenerational transmission of warm-sensitive-stimulating parenting: A prospective study of mothers and fathers of 3-year-olds. *Child Dev.* **76**, 384–396 (2005).
6. Fraser, A. *et al.* Cohort profile: The Avon longitudinal study of parents and children: ALSPAC mothers cohort. *Int. J. Epidemiol.* **42**, 97–110 (2013).
7. Boyd, A. *et al.* Cohort profile: The 'Children of the 90s'-The index offspring of the Avon longitudinal study of parents and children. *Int. J. Epidemiol.* **42**, 111–127 (2013).
8. Joshi, H. & Fitzsimons, E. The Millennium Cohort Study: The making of a multi-purpose resource for social science and policy. *Longit. Life Course Stud.* **7**, 409–430 (2016).
9. Connelly, R. & Platt, L. Cohort profile: UK Millennium Cohort Study (MCS). *Int. J. Epidemiol.* **43**, 1719–1725 (2014).
10. Sonnega, A. *et al.* Cohort profile: The Health and Retirement Study (HRS). *Int. J. Epidemiol.* **43**, 576–585 (2014).
11. Herd, P., Carr, D. & Roan, C. Cohort profile: Wisconsin Longitudinal Study (WLS). *Int. J. Epidemiol.* **43**, 34–41 (2014).
12. Howie, B. N., Donnelly, P. & Marchini, J. A flexible and accurate genotype imputation method for the next generation of genome-wide association studies. *Plos Genet.* **5**, e1000529 (2009).
13. Abecasis, G. R. *et al.* An integrated map of genetic variation from 1,092 human genomes. *Nature* **491**, 56–65 (2012).
14. Sherry, S. T. dbSNP: The NCBI database of genetic variation. *Nucleic Acids Res.* **29**, 308–311 (2001).
15. Fitzsimons, E. *et al. Collection of DNA samples and genetic data at scale in the UK Millennium Cohort Study.* (2020).
16. Laurie, C. C. *et al.* Quality control and quality assurance in genotypic data for genome-wide association studies. *Genet. Epidemiol.* **34**, 591–602 (2010).
17. McCarthy, S. *et al.* A reference panel of 64,976 haplotypes for genotype imputation. *Nat. Genet. 2016 4810* **48**, 1279–1283 (2016).
18. Lee, J. J. *et al.* Gene discovery and polygenic prediction from a genome-wide association study of educational attainment in 1.1 million individuals. *Nat. Genet.* **50**, 1112–1121 (2018).
19. Dudbridge, F. Power and predictive accuracy of polygenic risk scores. *PLoS Genet.* **9**,

e1003348 (2013).
20. Euesden, J., Lewis, C. M. & O'Reilly, P. F. PRSice: Polygenic Risk Score software. *Bioinformatics* **31**, 1466–1468 (2015).
21. Vilhjálmsson, B. J. *et al.* Modeling linkage disequilibrium increases accuracy of polygenic risk scores. *Am. J. Hum. Genet.* **97**, 576–592 (2015).
22. Turley, P. *et al.* Multi-trait analysis of genome-wide association summary statistics using MTAG. *Nat. Genet. 2018 502* **50**, 229–237 (2018).
23. Price, A. L. *et al.* Principal components analysis corrects for stratification in genome-wide association studies. *Nat. Genet.* **38**, 904–909 (2006).
24. Ward, C., Lewis, S. & Coleman, T. Prevalence of maternal smoking and environmental tobacco smoke exposure during pregnancy and impact on birth weight: retrospective study using Millennium Cohort. *BMC Public Heal. 2007 71* **7**, 1–7 (2007).
25. Wertz, J. *et al.* Using DNA from mothers and children to study parental investment in children's educational attainment. *Child Dev.* **91**, 1745–1761 (2020).
26. Bradley, R. H., Caldwell, B. M., Rock, S. L., Hamrick, H. M. & Harris, P. Home Observation for Measurement of the Environment: Development of a Home Inventory for use with families having children 6 to 10 years old. *Contemp. Educ. Psychol.* **13**, 58–71 (1988).
27. Caldwell, B. M. & Bradley, R. H. *Home observation for measurement of the environment.* (University of Arkansas at Little Rock, 1984).
28. Wertz, J. *et al.* Genetics of nurture: A test of the hypothesis that parents' genetics predict their observed caregiving. *Dev. Psychol.* **55**, 1461–1472 (2019).
29. National Institute of Child Health and Human Development Early Child Care Research Network. Child care and mother-child interaction in the first 3 years of life. *Dev. Psychol.* **35**, 1399–1413 (1999).
30. Magaña, A. B. *et al.* A brief method for assessing expressed emotion in relatives of psychiatric patients. *Psychiatry Res.* **17**, 203–212 (1986).
31. Caspi, A. *et al.* Maternal expressed emotion predicts children's antisocial behavior problems: using monozygotic-twin differences to identify environmental effects on behavioral development. *Dev. Psychol.* **40**, 149–61 (2004).
32. Robinson, E. A. & Eyberg, S. M. The dyadic parent–child interaction coding system: Standardization and validation. *J. Consult. Clin. Psychol.* **49**, 245–250 (1981).
33. Webster-Stratton, C. Preventing conduct problems in Head Start children: Strengthening parenting competencies. *J. Consult. Clin. Psychol.* **66**, 715–730 (1998).
34. Dunn, J., Deater-Deckard, K., Pickering, K., O'Connor, T. G. & Golding, J. Children's adjustment and prosocial behaviour in step-, single-parent, and non-stepfamily settings: Findings from a community study. *J. Child Psychol. Psychiatry* **39**, 1083–1095 (1998).
35. Johnson, J., Atkinson, M. & Rosenberg, R. *Millennium cohort study: Psychological, developmental and health inventories*. (2015).
36. Matheny, A. P., Wachs, T. D., Ludwig, J. L. & Phillips, K. Bringing order out of chaos: Psychometric characteristics of the confusion, hubbub, and order scale. *J. Appl. Dev. Psychol.* **16**, 429–444 (1995).
37. Jensen, E. W., James, S. A., Boyce, W. T. & Hartnett, S. A. The family routines inventory: Development and validation. *Soc. Sci. Med.* **17**, 201–211 (1983).
38. Fiese, B. H. & Kline, C. A. Development of the Family Ritual Questionnaire: Initial reliability and validation studies. *J. Fam. Psychol.* **6**, 290–299 (1993).

39. Evans, G. W., Gonnella, C., Marcynyszyn, L. a, Gentile, L. & Salpekar, N. The role of chaos in poverty and children's socioemotional adjustment. *Psychol. Sci.* **16**, 560–5 (2005).
40. Alati, R. *et al.* Effect of prenatal alcohol exposure on childhood academic Outcomes: Contrasting maternal and paternal associations in the ALSPAC study. *PLoS One* **8**, e74844 (2013).
41. Wertz, J. *et al.* Vital personality scores and healthy aging: Life-course associations and familial transmission. *Soc. Sci. Med.* **285**, 114283 (2021).
42. Northstone, K. & Emmett, P. M. Are dietary patterns stable throughout early and mid-childhood? A birth cohort study. *Br. J. Nutr.* **100**, 1069–1076 (2008).
43. Wertz, J. *et al.* Parental monitoring and knowledge: Testing bidirectional associations with youths' antisocial behavior. *Dev. Psychopathol.* **28**, 623–638 (2016).
44. Stattin, H. & Kerr, M. Parental monitoring: a reinterpretation. *Child Dev.* **71**, 1072–85 (2000).
45. StataCorp. *Stata Statistical Software: Release 17.* (StataCorp LLC, 2021).
46. Muthén, L. K. & Muthén, B. O. *Mplus User's Guide. Eighth Edition.* (Muthén & Muthén).
47. Kenny, D. A., Kashy, D. A. & Cook, W. L. *Dyadic data analysis*. (Guildford, 2006).
48. Ketende, S. C. & Jones, E. M. *User Guide to Analysing MCS Data Using STATA.* (2011).
49. Burke, H. *et al.* Prenatal and passive smoke exposure and incidence of asthma and wheeze: Systematic review and meta-analysis. *Pediatrics* **129**, 735–744 (2012).
50. Pereira, P. P. da S., Da Mata, F. A. F., Figueiredo, A. C. G., de Andrade, K. R. C. & Pereira, M. G. Maternal active smoking during pregnancy and low birth weight in the americas: A systematic review and meta-analysis. *Nicotine and Tobacco Research* **19**, 497–505 (2017).
51. Oken, E., Levitan, E. B. & Gillman, M. W. Maternal smoking during pregnancy and child overweight: Systematic review and meta-analysis. *Int. J. Obes.* **32**, 201–210 (2008).
52. Wakschlag, L. S., Pickett, K. E., Cook, E., Benowitz, N. L. & Leventhal, B. L. Maternal smoking during pregnancy and severe antisocial behavior in offspring: A review. *Am. J. Public Health* **92**, 966–974 (2002).
53. DiFranza, J. R., Aligne, C. A. & Weitzman, M. Prenatal and postnatal environmental tobacco smoke exposure and children's health. *Pediatrics* **113**, 1007–1015 (2004).
54. Rice, F., Langley, K., Woodford, C., Davey Smith, G. & Thapar, A. Identifying the contribution of prenatal risk factors to offspring development and psychopathology: What designs to use and a critique of literature on maternal smoking and stress in pregnancy. *Dev. Psychopathol.* **30**, 1107–1128 (2018).
55. Dasgupta, K., Ghimire, K. M. & Pacheco, G. How heavy is the price of smoking? Estimating the effects of prenatal smoking on child weight outcomes. *Appl. Econ.* **54**, 5159–5175 (2022).
56. Veisani, Y., Jenabi, E., Delpisheh, A. & Khazaei, S. Effect of prenatal smoking cessation interventions on birth weight: meta-analysis. *J. Matern. Neonatal Med.* **32**, 332–338 (2019).
57. Easey, K. E., Dyer, M. L., Timpson, N. J. & Munafò, M. R. Prenatal alcohol exposure and offspring mental health: A systematic review. *Drug Alcohol Depend.* **197**, 344–353 (2019).
58. Patra, J. *et al.* Dose-response relationship between alcohol consumption before and during

pregnancy and the risks of low birthweight, preterm birth and small for gestational age (SGA)-a systematic review and meta-analyses. *BJOG An Int. J. Obstet. Gynaecol.* **118**, 1411–1421 (2011).
59. Mamluk, L. *et al.* Evidence of detrimental effects of prenatal alcohol exposure on offspring birthweight and neurodevelopment from a systematic review of quasi-experimental studies. *Int. J. Epidemiol.* **49**, 1972–1995 (2020).
60. Yan, J., Liu, L., Zhu, Y., Huang, G. & Wang, P. P. The association between breastfeeding and childhood obesity: A meta-analysis. *BMC Public Health* **14**, 1–11 (2014).
61. Dogaru, C. M., Nyffenegger, D., Pescatore, A. M., Spycher, B. D. & Kuehni, C. E. Breastfeeding and childhood asthma: Systematic review and meta-Analysis. *Am. J. Epidemiol.* **179**, 1153–1167 (2014).
62. Horta, B. L., Loret De Mola, C. & Victora, C. G. Breastfeeding and intelligence: A systematic review and meta-analysis. *Acta Paediatr. Int. J. Paediatr.* **104**, 14–19 (2015).
63. Smithers, L. G., Kramer, M. S. & Lynch, J. W. Effects of breastfeeding on obesity and intelligence: Causal insights from different study designs. *JAMA Pediatr.* **169**, 707–708 (2015).
64. Anderson, N. J., Graham, S. A., Prime, H., Jenkins, J. M. & Madigan, S. Linking Quality and Quantity of Parental Linguistic Input to Child Language Skills: A Meta-Analysis. *Child Dev.* **92**, 484–501 (2021).
65. Han, J., Cui, N., Lyu, P. & Li, Y. Early-life home environment and child cognitive function: A meta-analysis. *Pers. Individ. Dif.* **200**, 111905 (2023).
66. Madigan, S. *et al.* Parenting behavior and child language: A Meta-analysis. *Pediatrics* **144**, (2019).
67. Hoeve, M. *et al.* The relationship between parenting and delinquency: a meta-analysis. *J. Abnorm. Child Psychol.* **37**, 749–75 (2009).
68. Pinquart, M. Associations of parenting styles and dimensions with academic achievement in children and adolescents: A Meta-analysis. *Educ. Psychol. Rev.* **28**, 475–493 (2016).
69. Marsh, S., Dobson, R. & Maddison, R. The relationship between household chaos and child, parent, and family outcomes: A systematic scoping review. *BMC Public Health* **20**, 1–27 (2020).
70. Andrews, K., Atkinson, L., Harris, M. & Gonzalez, A. Examining the effects of household chaos on child executive functions: A meta-analysis. *Psychol. Bull.* **147**, 16–32 (2021).
71. Madigan, S., McArthur, B. A., Anhorn, C., Eirich, R. & Christakis, D. A. Associations between screen use and child language skills: A systematic review and meta-analysis. *JAMA Pediatr.* **174**, 665–675 (2020).
72. Collett, B. R. *et al.* Observed child and parent toothbrushing behaviors and child oral health. *Int. J. Paediatr. Dent.* **26**, 184–192 (2016).
73. Fan, X. & Chen, M. Parental involvement and students' academic achievement: A meta-analysis. *Educ. Psychol. Rev.* **13**, 1–22 (2001).
74. Dowdall, N. *et al.* Shared picture book reading interventions for child language development: A systematic review and meta-analysis. *Child Dev.* **91**, e383–e399 (2020).
75. Noble, C. *et al.* The impact of shared book reading on children's language skills: A meta-analysis. *Educ. Res. Rev.* **28**, 100290 (2019).
76. Furlong, M. *et al.* Behavioural and cognitive-behavioural group-based parenting programmes for early-onset conduct problems in children aged 3 to 12 years. *Cochrane Database Syst. Rev.* (2012). doi:10.1002/14651858.cd008225.pub2

77. Barlow, J., Bergman, H., Kornor, H., Wei, Y. & Bennett, C. Group-based parent training programmes for improving emotional and behavioural adjustment in children. *Cochrane Database Syst. Rev. 2016,* CD003680 (2016). doi:10.1111/ijn.12540
78. Dretzke, J. *et al.* The clinical effectiveness of different parenting programmes for children with conduct problems: A systematic review of randomised controlled trials. *Child Adolesc. Psychiatry Ment. Health* **3**, (2009).
79. Jaffee, S. R., Hanscombe, K. B., Haworth, C. M. A., Davis, O. S. P. & Plomin, R. Chaotic homes and children's disruptive behavior: A longitudinal cross-lagged twin study. *Psychol. Sci.* **23**, 643–650 (2012).
80. Aliakbari, E. *et al.* Home-based toothbrushing interventions for parents of young children to reduce dental caries: A systematic review. *Int. J. Paediatr. Dent.* **31**, 37–79 (2021).
81. Martin, K. B., Bednarz, J. M. & Aromataris, E. C. Interventions to control children's screen use and their effect on sleep: A systematic review and meta-analysis. *J. Sleep Res.* **30**, 1–17 (2021).
82. Valcan, D. S., Davis, H. & Pino-Pasternak, D. Parental behaviours predicting early childhood executive functions: A meta-analysis. *Educ. Psychol. Rev.* **30**, 607–649 (2018).
83. Avvisati, F., Gurgand, M., Guyon, N. & Maurin, E. Getting parents involved: A field experiment in deprived schools. *Rev. Econ. Stud.* **81**, 57–83 (2014).
84. Dittus, P. J. *et al.* Parental monitoring and its associations with adolescent sexual risk behavior: A meta-analysis. *Pediatrics* **136**, e1587–e1599 (2015).
85. Allen, M. L. *et al.* Effective parenting interventions to reduce youth substance use: A systematic review. *Pediatrics* **138**, (2016).
86. Widman, L., Evans, R., Javidi, H. & Choukas-Bradley, S. Assessment of parent-based interventions for adolescent sexual health: A systematic review and meta-analysis. *JAMA Pediatr.* **173**, 866–877 (2019).
87. Boserup, S. H., Kopczuk, W. & Kreiner, C. T. The role of bequests in shaping wealth inequality: Evidence from Danish wealth records. *Am. Econ. Rev.* **106**, 656–661 (2016).
88. Spiteri, J. & von Brockdorff, P. Household Wealth and Inheritance Transfers: Evidence from the Euro Area. *J. Fam. Econ. Issues* (2022). doi:10.1007/s10834-022-09861-0
89. Druedahl, J. & Martinello, A. *Long-run saving dynamics: Evidence from unexpected inheritances*. (2017).
90. Thomese, F. & Liefbroer, A. C. Child care and child births: The role of grandparents in the Netherlands. *J. Marriage Fam.* **75**, 403–421 (2013).
91. Kanji, S. Grandparent Care: A key factor in mothers' labour force participation in the UK. *J. Soc. Policy* **47**, 523–542 (2018).
92. Posadas, J. & Vidal-Fernandez, M. Grandparents' childcare and female labor force participation. *IZA J. Labor Policy* **2**, 1–20 (2013).
93. Polderman, T. J. C. *et al.* Meta-analysis of the heritability of human traits based on fifty years of twin studies. *Nat. Genet.* **47**, 702–709 (2015).
94. Visscher, P. M. *et al.* 10 years of GWAS discovery: Biology, function, and translation. *Am. J. Hum. Genet.* **101**, 5–22 (2017).
95. Young, A. I., Benonisdottir, S., Przeworski, M. & Kong, A. Deconstructing the sources of genotype-phenotype associations in humans. *Science (80-.).* **365**, 1396–1400 (2019).
96. Howe, L. J. *et al.* Within-sibship genome-wide association analyses decrease bias in estimates of direct genetic effects. *Nat. Genet.* **54**, 581–592 (2022).
