# Supplementary material for: Genetic associations with parental investment from conception to wealth inheritance in six cohorts
Source: Nat Hum Behav. 2023 Jun 29;7(8):1388–401. doi: 10.1038/s41562-023-01618-5 (PMC10444618; doi:10.1038/s41562-023-01618-5)
Supplement: Supplementary file 2 — Reporting Summary [file 41562_2023_1618_MOESM2_ESM.pdf]

nature portfolio

Corresponding author(s): Jasmin Wertz

Last updated by author(s): Apr 14, 2023

Reporting Summary

Nature Portfolio wishes to improve the reproducibility of the work that we publish. This form provides structure for consistency and transparency in reporting. For further information on Nature Portfolio policies, see our Editorial Policies and the Editorial Policy Checklist.

Statistics

For all statistical analyses, confirm that the following items are present in the figure legend, table legend, main text, or Methods section.

n/a	Confirmed	
☐	☒	The exact sample size (*n*) for each experimental group/condition, given as a discrete number and unit of measurement
☐	☒	A statement on whether measurements were taken from distinct samples or whether the same sample was measured repeatedly
☐	☒	The statistical test(s) used AND whether they are one- or two-sided<br>*Only common tests should be described solely by name; describe more complex techniques in the Methods section.*
☐	☒	A description of all covariates tested
☐	☒	A description of any assumptions or corrections, such as tests of normality and adjustment for multiple comparisons
☐	☒	A full description of the statistical parameters including central tendency (e.g. means) or other basic estimates (e.g. regression coefficient) AND variation (e.g. standard deviation) or associated estimates of uncertainty (e.g. confidence intervals)
☐	☒	For null hypothesis testing, the test statistic (e.g. *F*, *t*, *r*) with confidence intervals, effect sizes, degrees of freedom and *P* value noted<br>*Give P values as exact values whenever suitable.*
☒	☐	For Bayesian analysis, information on the choice of priors and Markov chain Monte Carlo settings
☒	☐	For hierarchical and complex designs, identification of the appropriate level for tests and full reporting of outcomes
☒	☐	Estimates of effect sizes (e.g. Cohen's *d*, Pearson's *r*), indicating how they were calculated

Our web collection on statistics for biologists contains articles on many of the points above.

Software and code

Policy information about availability of computer code

Data collection	This study uses secondary data so we did not collect any data.
Data analysis	We did not use any software for collecting data. For constructing polygenic scores the softwares PRsice software v1.22, http://prsice.info/; Euesden, Lewis, & O'Reilly, 2015); LDPred (version 1.0.11) and PLINK v1.9 (Chang et al., 2015) were used. For analyising the data, Stata version 17.0 (StataCorp, 2021); Mplus version 8.2 (Muthén & Muthén, 1998-2017); R version 4.2.1 (2022-06-23). All the code is available on request.

For manuscripts utilizing custom algorithms or software that are central to the research but not yet described in published literature, software must be made available to editors and reviewers. We strongly encourage code deposition in a community repository (e.g. GitHub). See the Nature Portfolio guidelines for submitting code & software for further information.

Data

Policy information about availability of data

All manuscripts must include a data availability statement. This statement should provide the following information, where applicable:

- Accession codes, unique identifiers, or web links for publicly available datasets
- A description of any restrictions on data availability
- For clinical datasets or third party data, please ensure that the statement adheres to our policy

MCS phenotypic data are available for free via the UK Data Service (https://ukdataservice.ac.uk/2020/10/14/millennium-cohort-study-age-17-data-now-available/); MCS genetic data are available for free, through managed access via the UCL Centre for Longitudinal Studies Data Access Committee (https://cls.ucl.ac.uk/data-access-training/data-access/accessing-data-directly-from-cls/). ALSPAC phenotypic and genetic data are available for a fee, through managed access via the ALSPAC Executive Committee (http://www.bristol.ac.uk/alspac/researchers/access/). E-Risk and Dunedin phenotypic and genetic data are available for free, through managed access via the respective study units (https://sites.duke.edu/moffittcaspiprojects/data-use-guidelines/). HRS phenotypic and genetic polygenic-score data

are available for free via the HRS study website (https://hrs.isr.umich.edu/data-products). WLS genotypic data are available for free via the WLS study website (https://www.ssc.wisc.edu/wlsresearch/data/); WLS genetic data are available for free, through managed access via the WLS PIs (https://www.ssc.wisc.edu/wlsresearch/documentation/GWAS/).

Field-specific reporting

Please select the one below that is the best fit for your research. If you are not sure, read the appropriate sections before making your selection.

☐ Life sciences ☒ Behavioural & social sciences ☐ Ecological, evolutionary & environmental sciences

For a reference copy of the document with all sections, see nature.com/documents/nr-reporting-summary-flat.pdf

Behavioural & social sciences study design

All studies must disclose on these points even when the disclosure is negative.

Study description	This study uses quantitative data.
Research sample	We used the following existing datasets: MCS cohort (n=6,732), based in the UK, nationally representative when weighted; ALSPAC cohort (n=7,588) based in the UK, not nationally representative; E-Risk cohort (n=880), based in the UK, nationally representative; Dunedin cohort (n=643), based in New Zealand, nationally representative; HRS cohort (n=8,652), based in the US, nationally representative; WRS cohort (n=8,479), based in the US, not nationally representative. Each sample was chose because it had measures of genetics in parents; measures of parenting; and a sample size of at least 200 individuals. Sources for the data are available in the links in the “Data availability” statement above.
Sampling strategy	We did not collect any data, so did not use a sampling strategy. In the original cohort recruitment, the following sampling strategies were used: MCS=stratified and clustered sampling; ALSPAC=opportunity sampling; E-Risk=stratified sampling; Dunedin=stratified sampling; HRS=stratified sampling; WLS=stratified sampling. Because we did not collect data, we did not predetermine sample sizes. However, we set out to include cohorts that would have at least 200 participants because of power calculations indicating that we would need at least n=200 individuals to be able to detect correlations of r=.20. All our sample sizes exceed this limit, often substantially so.
Data collection	We did not collect any data for this study. Instruments used in the original data collection were paper questionnaires, postal questionnaires, and online questionnaires. The interviewers collecting the data were blind to the study’s hypotheses, in the sense that they were unaware of the study questions the data would be used for (because the data were collected before the research questions in this study had been developed).
Timing	For the MCS, data were collected between 2001 and 2019. For ALSPAC, data were collected between 1991 and 2010. For E-Risk, data were collected between 1998 and 2007. For Dunedin, data were collected between 1994 and 2019. For HRS, data were collected between 1992 and 2016. For WLS, data were collected between 1957 and 2011.
Data exclusions	In MCS and ALSPAC we excluded families with multiples (n=158 in MCS and n=184 in ALSPAC). Otherwise we did not make any exclusions of individuals who had data in our study variables.
Non-participation	For MCS, by age 17 (the last assessment age we included), n=8061 of the original sample had dropped out of the sample due to refusal to participate or inability to trace the participant. For ALSPAC, by age 17 (the last assessment age we included), n=7141 of the original sample had dropped out of the sample due to refusal to participate or inability to trace the participant. For E-Risk, by age 12 (the last assessment age we included), n=43 of the original n=1,116 had dropped out of the sample due to refusal to participate or inability to trace the participant. For Dunedin, there was only one assessment wave, so there was no dropout over time. For HRS and WLS, we used data from a minimum of one assesment wave, so people were included even if they dropped out at later assessment.
Randomization	This was not an experiment, so we did not assign people to experimental conditions (randomly or otherwise).

Reporting for specific materials, systems and methods

We require information from authors about some types of materials, experimental systems and methods used in many studies. Here, indicate whether each material, system or method listed is relevant to your study. If you are not sure if a list item applies to your research, read the appropriate section before selecting a response.

Materials & experimental systems

n/a	Involved in the study
☒	☐ Antibodies
☒	☐ Eukaryotic cell lines
☒	☐ Palaeontology and archaeology
☒	☐ Animals and other organisms
☐	☒ Human research participants
☒	☐ Clinical data
☒	☐ Dual use research of concern

Methods

n/a	Involved in the study
☒	☐ ChIP-seq
☒	☐ Flow cytometry
☒	☐ MRI-based neuroimaging

Human research participants

Policy information about studies involving human research participants

Population characteristics	This information is provided in the manuscript for each cohort.
Recruitment	This information is provided in the manuscript for each cohort.
Ethics oversight	This information is provided in the manuscript for each cohort.

Note that full information on the approval of the study protocol must also be provided in the manuscript.
